# The impact of organic extracts of seasonal PM_2.5_ on primary human lung epithelial cells and their chemical characterization

**DOI:** 10.1007/s11356-021-14850-1

**Published:** 2021-06-20

**Authors:** Jieun Park, Kyoung-Hee Lee, Hyewon Kim, Jisu Woo, Jongbae Heo, Chang-Hoon Lee, Seung-Muk Yi, Chul-Gyu Yoo

**Affiliations:** 1 Graduate School of Public Health, Seoul National University, Seoul, Korea; 2 Division of Pulmonary and Critical Care Medicine, Department of Internal Medicine, Seoul National University Hospital, 101 Daehakno, Jongno-gu, Seoul, 03080 Korea; 3 Busan Development Institute, 955 Jungangdae-ro, Busanjin-gu, Busan, 47210 Korea; 4 Department of Internal Medicine, Seoul National University College of Medicine, Seoul, Korea; 5 Institute of Health and Environment, Seoul National University, Seoul, Korea

**Keywords:** PM_2.5_, Organic compounds, Lung epithelial cells, Cytokine, Senescence, Macroautophagy

## Abstract

Lung epithelial cells serve as the first line of defense against various inhaled pollutant particles. To investigate the adverse health effects of organic components of fine particulate matter (PM_2.5_) collected in Seoul, South Korea, we selected 12 PM_2.5_ samples from May 2016 to January 2017 and evaluated the effects of organic compounds of PM_2.5_ on inflammation, cellular aging, and macroautophagy in human lung epithelial cells isolated directly from healthy donors. Organic extracts of PM_2.5_ specifically induced neutrophilic chemokine and interleukin-8 expression via extracellular signal-regulated kinase activation. Moreover, PM_2.5_ significantly increased the expression of aging markers (p16, p21, and p27) and activated macroautophagy. Average mass concentrations of organic and elemental carbon had no significant correlations with PM_2.5_ effects. However, polycyclic aromatic hydrocarbons and n-alkanes were the most relevant components of PM_2.5_ that correlated with neutrophilic inflammation. Vegetative detritus and residential bituminous coal combustion sources strongly correlated with neutrophilic inflammation, aging, and macroautophagy activation. These data suggest that the chemical composition of PM_2.5_ is important for determining the adverse health effects of PM_2.5_. Our study provides encouraging evidence to regulate the harmful components of PM_2.5_ in Seoul.

**Supplementary Information:**

The online version contains supplementary material available at 10.1007/s11356-021-14850-1.

## Introduction

The persistent occurrence of ambient air pollution has attracted considerable attention as a global environmental issue. The International Agency for Research on Cancer (IARC) classified particulate matter from outdoor air pollution as a Group 1 carcinogen in 2013 (Loomis et al. 2013). In particular, ambient fine particulate matter (PM_2.5_), which has an aerodynamic diameter of 2.5 μm or less, is correlated with an increase in mortality and morbidity caused by cardiovascular and pulmonary impairments (Davel et al. 2012; Bell et al. 2014; Tsai et al. 2013; Shah et al. 2015; Feng et al. 2016). Since the pulmonary airway is the first line of defense against inhaled PM_2.5_, studies have discovered that particulate matter induces oxidative stress and inflammation, causing inflammatory lung diseases, such as chronic obstructive pulmonary disease (COPD) and lung cancer (Pope and Dockery 1999; Donaldson et al. 2003; Anenberg et al. 2010; Kloog et al. 2013). Potential mechanisms underlying PM_2.5_-induced adverse health effects on the human respiratory system have been consistently reported in toxicological, experimental-based studies as well as epidemiological studies (Bell et al. 2014; Gualtieri et al. 2011; Lu et al. 2015; Xing et al. 2016). To investigate the effects of PM_2.5_, numerous toxicological studies have used commercial lung epithelial cells (Rumelhard et al. 2007; Alessandria et al. 2014; Cachon et al. 2014; Song et al. 2017) and Standard Reference Material (SRM) urban particulate matter. However, the effects of ambient particulate matter, collected in Seoul, South Korea, on primary human airway epithelial cells (HAECs) isolated directly from healthy donors have not been studied.

Due to the complexity of PM_2.5_ itself, the adverse health effects of PM_2.5_ may vary depending on its chemical characteristics, sources, and regions. While PM_2.5_ is composed of various chemical constituents, organic components comprise about 20–40% of PM_2.5_ mass in urban areas (He et al. 2001; Dan et al. 2004; Putaud et al. 2010). The concentrations of organic carbon (OC) and elemental carbon (EC) are highly correlated with adverse health effects, such as cardiopulmonary diseases, which require emergency hospitalization (Lanki et al. 2006; Vedal et al. 2013; Qiao et al. 2014). Additionally, organic compounds, such as polycyclic aromatic hydrocarbons (PAHs), are prominent carcinogens (Baird et al. 2005; Gilli et al. 2007; Dilger et al. 2016). Thus, finding the sources of PM_2.5_ based on the local chemical characteristics and linking them to toxicological effects is necessary. Assuming that PM_2.5_ in Seoul has distinct organic components and contributing sources, we analyzed organic compounds in PM_2.5_ and identified potential contributing sources using a receptor model. Recently, the frequency of high concentration events (HCEs) has been increasing in Seoul. According to *The 2016 Environmental Performance Index Report*, more than 50% of the Korean population is exposed to dangerous levels of PM_2.5_ (Hsu 2016). In the present study, we investigated the impact of organic extracts of PM_2.5_ collected in Seoul, South Korea, on primary human lung epithelial cells and identified the relevant components and sources in PM_2.5_.

## Methods

### Sampling site and collection procedure

PM_2.5_ samples were collected on the rooftop of the Graduate School of Public Health building (37.581̊N, 127.001̊E) at Seoul National University in Seoul, Korea. Samples were collected for 24 h using a high-volume air sampler and a low-volume air sampler equipped with a filter pack (URG-2000-30FG, URG, Chapel Hill, NC, USA) and cyclone (URG-2000-30EH, URG, USA). A high-volume air sampler loaded with quartz microfiber filters (Whatman^TM^, Maidstone, UK) collected PM_2.5_ at a flow rate of 40 cfm, and the collected filters were used for organic extraction. A low-volume air sampler was loaded with Teflon filters (PTFE membrane, Pall Corporation, USA) to measure mass concentrations, and quartz filters (Quartz microfiber filter, Pall Corporation, USA) to quantify OC and EC concentrations. The PM_2.5_ mass concentration was measured with a semimicro balance (accuracy of 0.01 mg) (CP225D, Sartorius, Goettingen, Germany), and 12 samples collected during HCEs between May 2016 and January 2017 were selected. Three HCE samples from each season were selected based on the Korean national air quality standards of PM_2.5_, i.e., a 24-h average concentration of 35 μg/m^3^. Thus, 12 HCE samples were used in this study.

### Organic extraction of the collected PM_2.5_ samples

Quartz filters were baked in a furnace at 450 °C for 24 h, and the collected filters were stored at – 20 °C until further use. Samples were punched using a stainless cutter, and two of the punched filters (4 cm × 4 cm) were used for the extraction. Solvent mixture of dichloromethane:methanol (3:1, v/v) was used for sample extractions with an ultrasonic bath. The extracted samples were concentrated to 10 mL using a Turbovap II (Zymark Co., USA) with N_2_ gas, and 0.2-μm Acrodisc Syringe Filters (Pall Corporation, USA) were used for filtration. The filtered samples were then concentrated to 1 mL using a Turbovap II and Reacti-Therm (Thermo Fisher Scientific, USA) under a gentle stream of N_2_ gas and were stored at – 20 °C. The concentrated samples were used for organic compound analysis and *in vitro* experiments.

### Cells and exposure protocol

Normal human bronchial epithelial cells (BEAS-2B from ATCC, Manassas, VA, USA) were maintained in defined keratinocyte-SFM (Gibco by Thermo Fisher Scientific, Waltham, MA, USA) at 37 °C under 5% CO_2_. Normal primary HAECs were obtained after review and approval by the Seoul National University Hospital Institutional Review Board (SNUH IRB number: H-1602-108-742). Primary HAECs were isolated from bronchial brushing samples during bronchoscopy. The brush was immediately immersed in a tube containing 10 mL of ice-cold RPMI supplemented with 20% fetal bovine serum. Within a few minutes, the cells were centrifuged and resuspended in defined keratinocyte-SFM. Submerged cells were grown as monolayers to 80–100% confluence and then used for experiments at passage no. 2. Rabbit polyclonal anti-phospho-p44/42 MAPK (Thr202/Tyr204) (p-ERK), anti-ERK, and anti-light chain 3B (LC3B) antibodies, and U0126 (a highly selective inhibitor of MEK1 and MEK2) were obtained from Cell Signaling (Danvers, MA, USA). Goat polyclonal anti-GAPDH and rabbit polyclonal anti-p21 and anti-p27 antibodies were purchased from Santa Cruz Biotechnology (Dallas, TX, USA). Rabbit monoclonal anti-p16 antibody was obtained from Abcam (Cambridge, MA, USA). Thiazolyl blue tetrazolium bromide (MTT) was purchased from Millopore Sigma (St. Louis, MO, USA). Both BEAS-2B cells and verified HAECs were treated with vehicle control or various concentrations of PM_2.5_ organic extracts (% v/v in culture media) for 0, 3, 6, or 24 h.

### Cell viability

Cell viability was measured using MTT and lactate dehydrogenase (LDH) release assays. MTT solution was added to the culture medium of cells (1 × 10^5^ cells/mL) (final concentration of MTT in the medium was 0.5 mg/mL), and the cells were incubated at 37 °C for 1 h (Lee et al. 2015). After removing the culture medium, 50 μL of DMSO (purity ≥ 99.9%) was added, and the optical density of each well was measured at 570 nm. LDH release assays were performed using a CytoTox-ONE^TM^ Homogeneous Membrane Integrity Assay Kit (Promega, Madison, WI, USA) according to the manufacturer’s instructions.

### Protein extraction and western blot analysis

Total cellular extracts were prepared in ice cold 1X cell lysis buffer (Cell Signaling). Equal amounts of protein were resolved using gradient SDS-polyacrylamide gel electrophoresis (Thermo Fisher Scientific, Waltham, MA, USA) and transferred to nitrocellulose membranes (Thermo Fisher Scientific). The membranes were blocked with 5% skim milk blocking buffer for 1 h before overnight incubation at 4 °C with primary antibodies. The membranes were then washed three times with washing buffer and incubated with horseradish peroxidase-conjugated secondary antibodies in blocking buffer for 1 h. After successive washes, the membranes were developed using a SuperSignal West Pico Chemiluminescent Kit (Thermo Fisher Scientific) (Lee et al. 2017).

### Multiplex bead assay

The levels of cytokines in cell culture media were determined using a Bio-Plex Pro^TM^ Cytokine Assay Kit (Bio-Rad, Hercules, CA, USA) according to the manufacturer’s instructions. Briefly, 50 μL of 1X antibodies coupled to magnetic beads was added to 96-well plates, following which the plates were washed twice. Fifty microliters of standards and samples (cell supernatants) was added to the plates and incubated for 1 h at room temperature (RT) with constant shaking at 850 rpm. The magnetic beads were washed three times. Then, detection antibodies (25 μL) were added and the samples were incubated for 30 min at RT with constant shaking at 850 rpm. The beads were washed three times. Streptavidin-PE (50 μL) was added to the plates and then incubated for 10 min at RT with constant shaking at 850 rpm. Following a wash, the beads were resuspended in 125 μL of assay buffer, shaken for 30 s at 850 rpm, and analyzed using the Bio-Plex system.

### Gas chromatography-mass spectrometry analysis and OC/EC analysis

Gas chromatography-mass spectrometry (7080B/5977B, Agilent Technologies, Inc., USA) was employed to quantify 52 organic compounds in each extract. The analyzed species included 23 species of PAHs, 17 species of n-alkanes, 7 species of hopanes, and 5 species of alkylcyclohexanes and isoprenoids.

The samples collected in the low-volume sampler were punched (1.5 cm × 1.0 cm) to analyze the major components of carbon species: OC and EC. OC and EC were analyzed using a carbon aerosol analyzer (Sunset Laboratory Inc., USA). Thermal/optical transmittance method was used for data quantification.

### Source apportionment of organic compounds in PM_2.5_ using chemical mass balance model

Source apportionment of the OC fraction of PM_2.5_ was performed using a chemical mass balance (CMB) (EPA-CMB v8.2) model provided by the U.S. Environmental Protection Agency (EPA). The CMB air quality model is a receptor model that has been widely used to identify sources and quantify source contributions (Coulter 2004). The concentrations of organic compounds, OC, and EC in the 12 samples were used as ambient data in addition to the speciated source profile data (Table S1). The optimal set of source profiles contained four sources: vegetative detritus (Rogge et al. 1993), residential bituminous coal combustion soot (Zhang et al. 2008), diesel engines (Lough et al. 2007), and gasoline motor vehicles (Lough et al. 2007).

### Statistical analyses

A two-tailed unpaired *t-test* was used to assess the statistical differences between groups, and statistical analyses were performed using GraphPad Prism software (San Diego, CA, USA). Correlations between chemical constituents/source contributions and cytokine production and expression of aging and macroautophagy markers were analyzed with Spearman correlation using R 3.4.0. Statistical significance was set at p-value < 0.05.

## Results

### Effect of PM_2.5_ organic compounds on cell viability in lung epithelial cells (BEAS-2B)

**Fig. 1:**
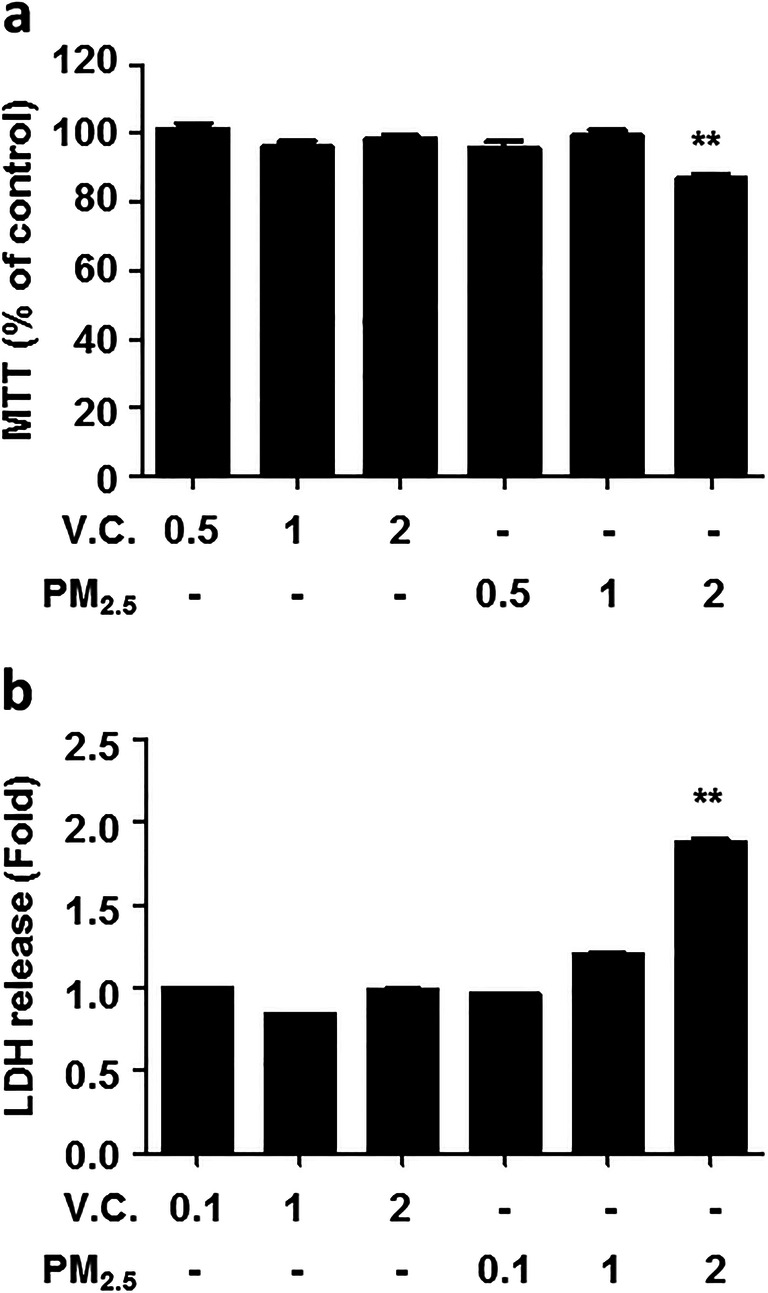
The effects of PM_2.5_ on cell viability in lung epithelial cells. BEAS-2B cells were exposed to V.C .or PM_2.5_ organic compounds (0.1, 0.5, 1, 2%) for 24 h. MTT (**a**) and LDH release assays (**b**) were performed. Data are presented as the mean ± SE. **p < 0.05

Since PM_2.5_ organic compounds have been shown to be cytotoxic, we first evaluated the dose-dependent effect of PM_2.5_ organic compounds on the viability of lung epithelial cells. BEAS-2B cells were treated with vehicle control (V.C.; dichloromethane) and PM_2.5_ organic extracts (0.1, 0.5, 1, and 2%) of a single sample (sample collected on November 8, 2016) for 24 h, and cell viability assays (MTT and LDH release assays) were performed. PM_2.5_ organic extracts having a concentration of 1% or less did not affect cell viability (Fig. 1). Based on this result, we used PM_2.5_ (1%) in all experiments.

### Effect of PM_2.5_ organic compounds on cytokine production and the expression of aging and macroautophagy markers in BEAS-2B cells

**Fig. 2:**
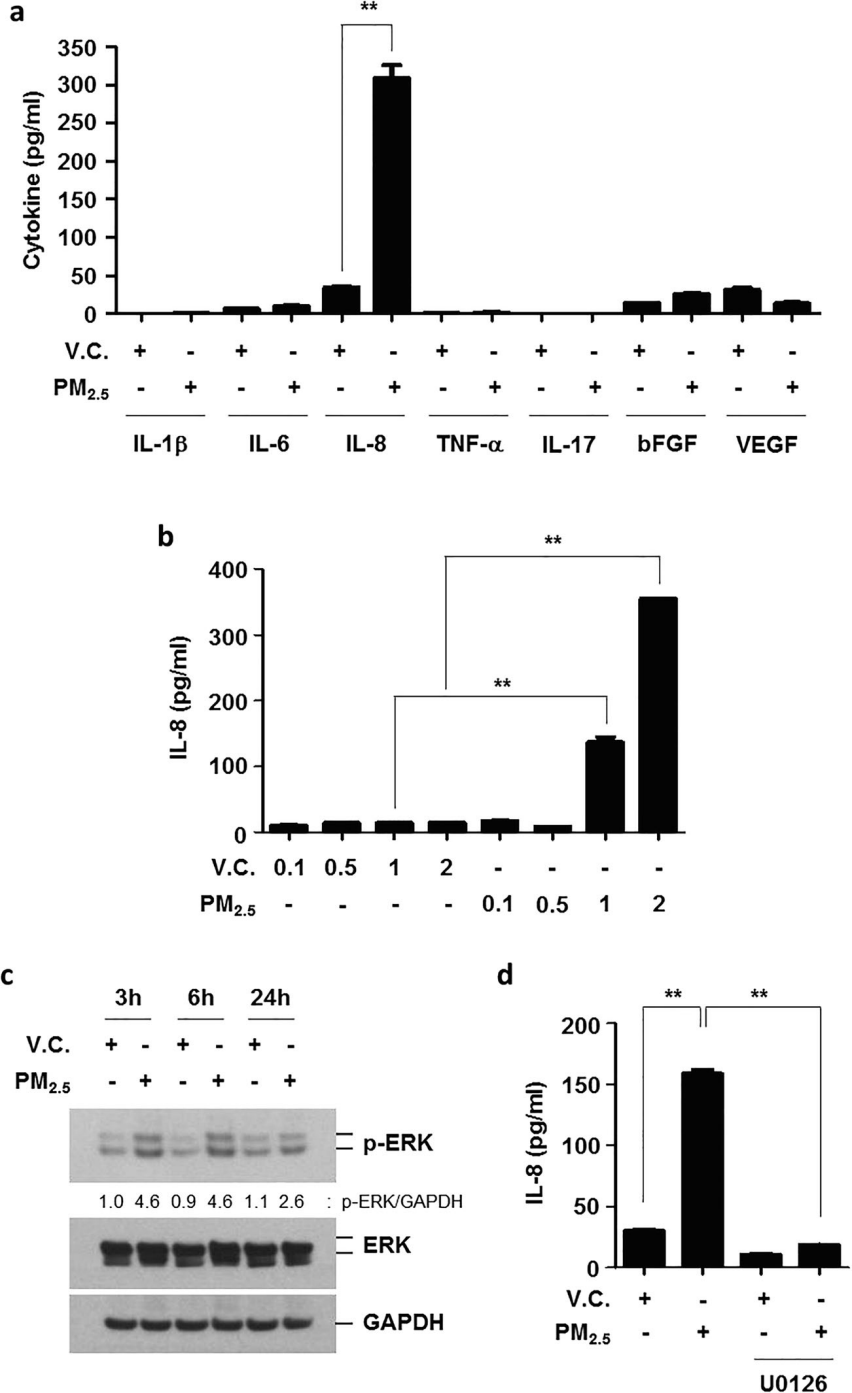
The effects of PM_2.5_ on cytokine production in BEAS-2B cells. **a** BEAS-2B cells were treated with V.C. or PM_2.5_ organic compounds (1%) for 24 h. **b** Cells were incubated with various concentrations (0.1, 0.5, 1, 2%) of V.C. or PM_2.5_ for 24 h. The levels of cytokines (IL-1β, IL-6, IL-8, TNF-α, -IL-17, bFGF, and VEGF) in culture media were measured by a multiplex bead assay. Data are presented as the mean ± SE. **p < 0.05. **c** BEAS-2B cells were treated with V.C. or PM_2.5_ (1%) for the indicated times. Total cellular extracts were subjected to western blot analysis for p-ERK, ERK, and GAPDH. Densitometry analysis was performed using Scion image software. **d** Cells were pretreated with U0126 (4 μM) for 2 h and then stimulated with V.C. or PM_2.5_ organic compounds (1%) for 24 h in the presence or absence of U0126. The level of IL-8 in media was measured by a multiplex bead assay. Data are presented as the mean ± SE. **p < 0.05

PM_2.5_ organic compounds exclusively induced the production of IL-8 but not of IL-1β, IL-6, TNF-α, IL-17, bFGF, or VEGF (Fig. 2a, b). Therefore, we investigated the role of the extracellular signal-regulated kinase (ERK) pathway in PM_2.5_-induced IL-8 production. PM_2.5_ activated the ERK pathway (Fig. 2c), and blocking ERK activation using a chemical inhibitor (U0126) decreased PM_2.5_-mediated IL-8 production (Fig. 2d). These data suggest that the ERK pathway is responsible for IL-8 release in response to PM_2.5_ stimulation of lung epithelial cells.

**Fig. 3:**
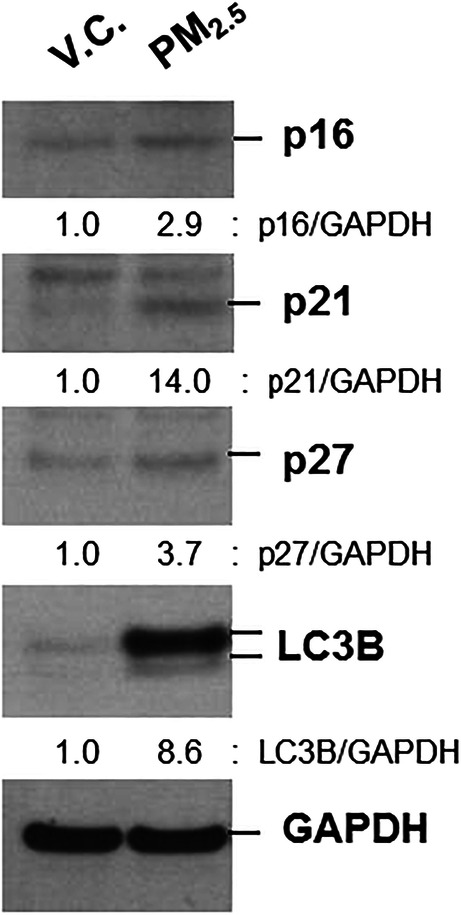
The effects of PM_2.5_on the expression of aging and macroautophagy markers in BEAS-2B cells. Cells were exposed to V.C. or PM_2.5_ organic compounds (1%) for 24 h. Total cell lysates were extracted and then subjected to western blot analysis for p16, p21, p27, LC3B, and GAPDH. Densitometry analysis was performed using Scion image software

Additionally, a significant increase in the expression levels of senescence markers (p16, p21, and p27) and macroautophagy marker (LC3B) was observed (Fig. 3).

### Effect of PM_2.5_ organic compounds on inflammation, aging, and macroautophagy activation in primary HAECs

**Fig. 4:**
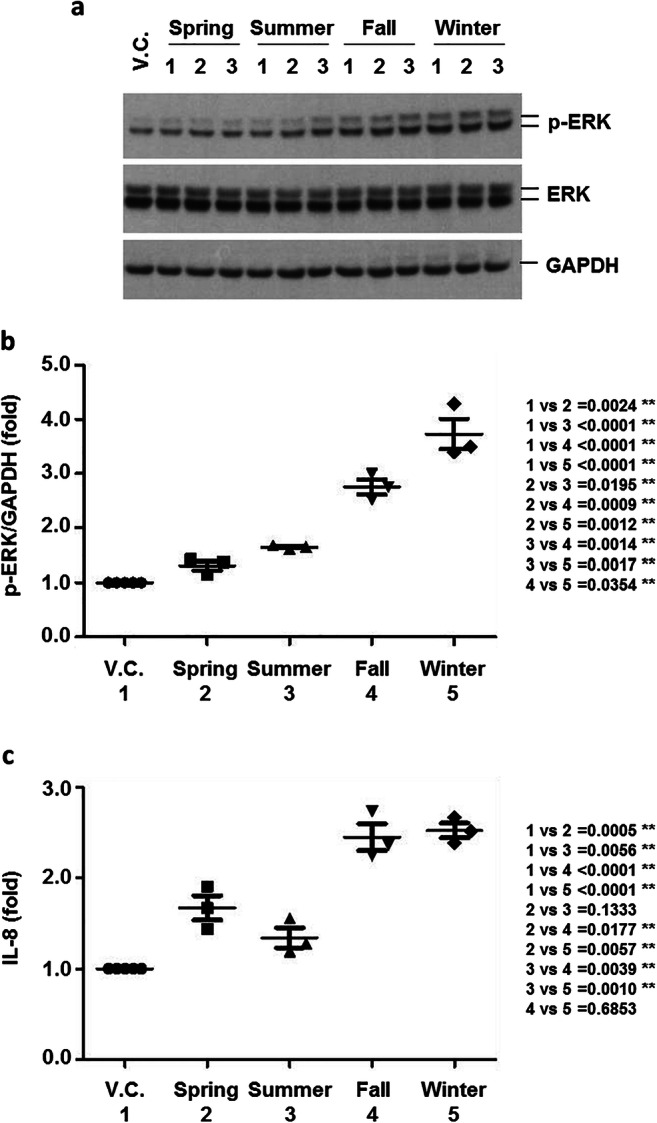
The effects of PM_2.5_ on ERK activation and IL-8 production in primary HAECs. Primary HAECs (n = 6) were exposed to V.C. or PM_2.5_ organic compounds (1%) for 24 h. Total cell lysates were extracted and then subjected to Western blot analysis for p-ERK, ERK, and GAPDH (**a**). Densitometry analysis using Scion image software for p-ERK. Blots were normalized to GAPDH expression (**b**). The IL-8 concentration in the cell culture media was measured using a multiplex bead assay (n = 5) (**c**). Data are presented as the mean ± SE. **p < 0.05

**Fig. 5:**
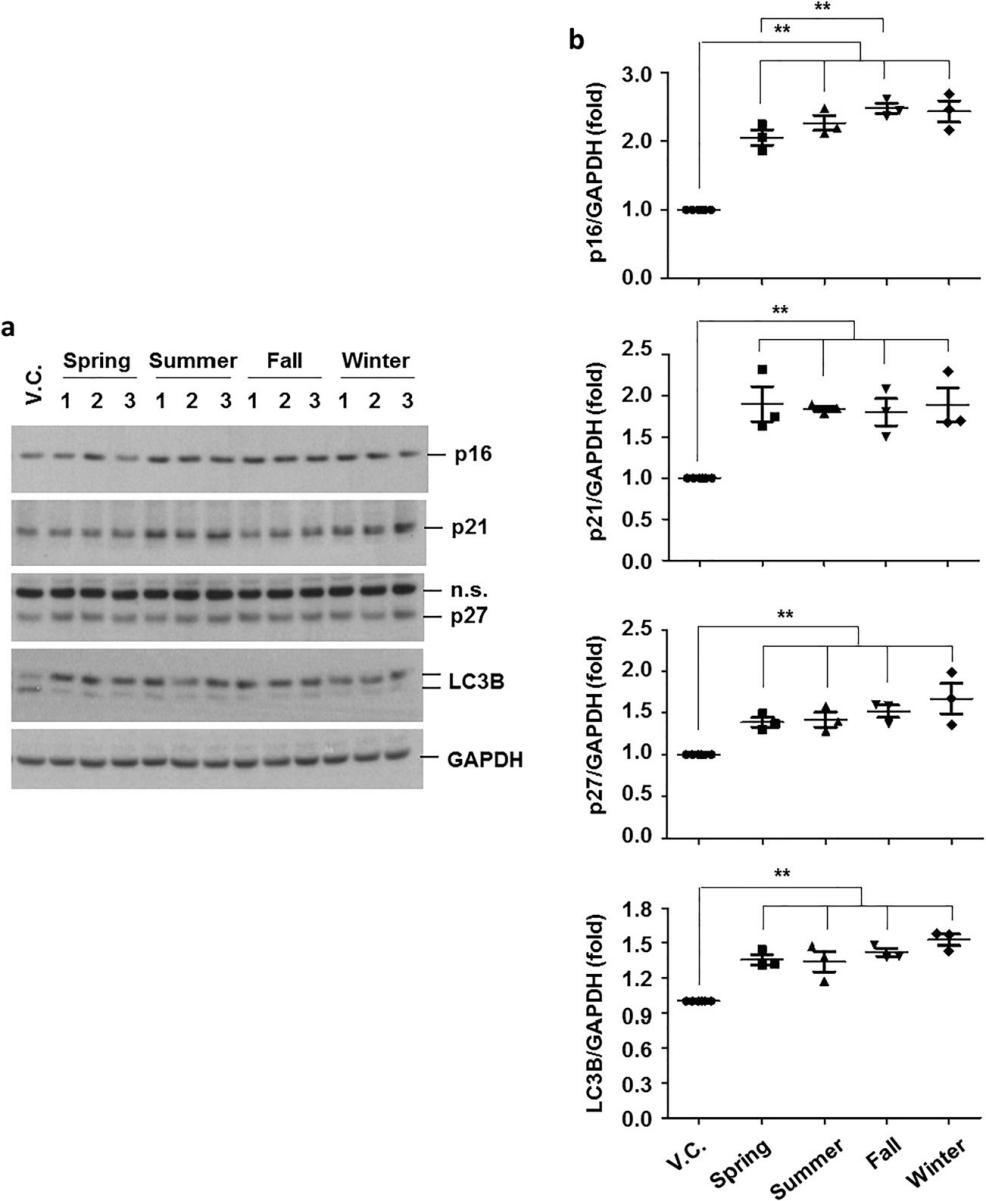
The effects of PM_2.5_ on the expression of aging and autophagy markers in primary HAECs. Primary HAECs (n = 6) were exposed to V.C. or PM_2.5_ organic compounds (1%) for 24 h. Total cell lysates were extracted and then subjected to Western blot analysis for p16, p21, p27, LC3B, and GAPDH (**a**). Densitometry analysis using Scion image software (**b**). Data are presented as the mean ± SE. **p < 0.05

To confirm the activation of the ERK pathway and increased levels of IL-8 in primary cells, primary HAECs from six healthy control patients with no symptoms of COPD or respiratory diseases were used for exposure analysis. PM_2.5_ activated the ERK pathway and induced IL-8 production (Fig. 4a–c). The expression levels of active ERK and IL-8 were significantly higher in cells exposed to fall and winter samples than in those exposed to spring and summer samples (Fig. 4b, c). Moreover, we observed that PM_2.5_ significantly increased the expression levels of senescence markers (p16, p21, and p27) and activated macroautophagy (Fig. 5a–c). No significant seasonal differences were found in the expression levels of senescence and macroautophagy markers (Fig. 5b).

### Analysis of PM_2.5_ constituents correlated with inflammation, aging, and macroautophagy activation

**Fig. 6:**
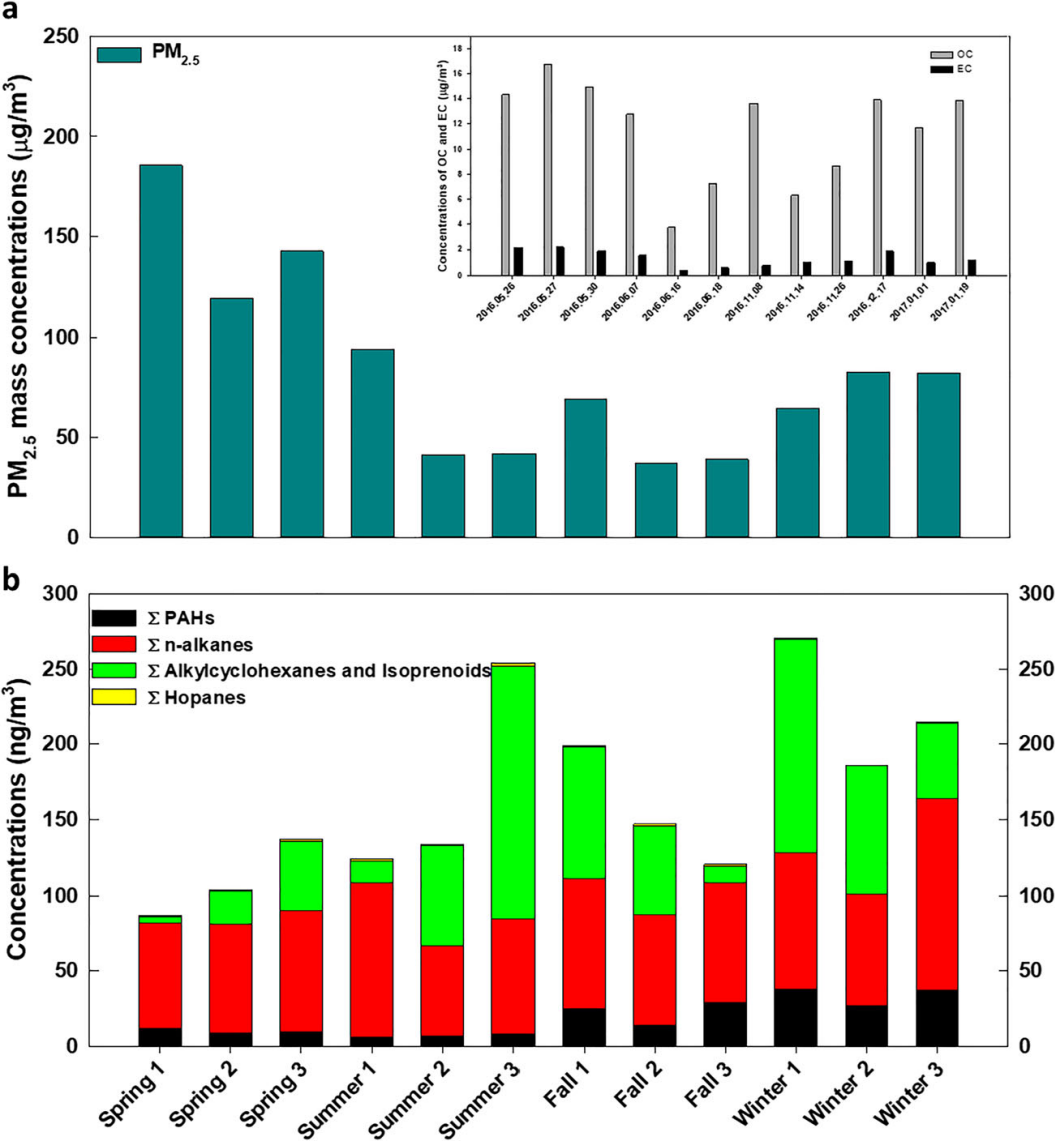
PM_2.5_ mass concentrations and concentrations of organic compounds. **a** PM_2.5_ mass concentrations and OC and EC concentrations in twelve samples. **b** Concentrations of organic compounds, including PAHs, n-alkanes, alkylcyclohexanes and isoprenoids, and hopanes

As shown in Fig. 6a, the average mass concentration of the 12 PM_2.5_ samples was 83.2 ± 3.85 μg/m^3^. When categorized seasonally, the highest average PM_2.5_ mass concentration was observed in spring (149 ± 11.2 μg/m^3^), followed by winter (76.4 ± 3.41 μg/m^3^), summer (59.0 ± 10.1 μg/m^3^), and fall (48.4 ± 5.97 μg/m^3^). The average concentrations of OC and EC in the 12 samples were 11.5 ± 0.34 μg/m^3^ and 1.32 ± 0.05 μg/m^3^, respectively. The seasonal averages of OC and EC concentrations and the seasonal average PM_2.5_ mass concentrations showed a similar trend. Thus, the average concentrations were the highest in the spring (OC 15.3 ± 0.43 μg/m^3^, EC 2.06 ± 0.05 μg/m^3^), followed by winter (OC 13.2 ± 0.41 μg/m^3^, EC 1.38 ± 0.14 μg/m^3^), fall (OC 9.53 ± 1.25 μg/m^3^, EC 1.00 ± 0.06 μg/m^3^) and summer (OC 7.92 ± 1.51 μg/m^3^, EC 0.84 ± 0.20 μg/m^3^).

The overall average and seasonal average concentrations of the sum of PAHs, n-alkanes, hopanes, and alkylcyclohexanes and isoprenoids were calculated and are presented in Fig. 6b (Table S3). While n-alkanes had the highest average concentrations among organic compounds, the highest average concentration was observed in winter (96.9 ± 9.05 ng/m^3^), followed by fall (80.1 ± 2.15 ng/m^3^), summer (79.5 ± 7.18 ng/m^3^), and spring (74.4 ± 1.75 ng/m^3^). The average concentrations of alkylcyclohexanes and isoprenoids were the highest in winter (92.1 ± 15.4 ng/m^3^) and the lowest in spring (23.8 ± 6.97 ng/m^3^). For PAHs, the average concentration in winter (34.0 ± 2.00 ng/m^3^) was the highest, followed by fall (22.3 ± 2.65 ng/m^3^), spring (9.88 ± 0.44 ng/m^3^), and summer (7.13 ± 0.37 ng/m^3^). Unlike other organic compounds, hopanes had the highest average concentration in summer (1.57 ± 0.13 ng/m^3^), followed by fall (1.25 ± 0.09 ng/m^3^), spring (1.04 ± 0.03 ng/m^3^), and winter (0.69 ± 0.12 ng/m^3^). The seasonal trends of organic compounds did not follow those of PM_2.5_ and OC.

The association between PM_2.5_ organic compounds and IL-8 production was measured using the Pearson correlation coefficient (r). R values greater than 0.70 with a p-value less than 0.05 indicated highly correlated compounds. PM_2.5_ mass concentrations, OC, and EC had negative or no significant correlations with inflammation, aging, and macroautophagy activation, unlike several organic compounds that showed significant correlations.

The results showed that increases in the levels of PAHs and several n-alkanes were highly correlated with increases in both ERK activation and IL-8 production (Table S4, Table S5). PAHs, such as phenanthrene, anthracene, fluoranthene, pyrene, cyclopenta[cd]pyrene, benzo[a]anthracene, benzo[b]fluoranthene, benzo[k]fluoranthene, benzo[a]pyrene, benzo[e]pyrene, indeno[1,2,3-cd]pyrene, dibenzo[a,h]anthracene, picene, benzo[ghi]perylene, and coronene showed a high correlation with active ERK and IL-8 expression levels. The n-alkanes showing high correlations with active ERK and IL-8 included C27, C30, C31, C32, C33, and C34. Among alkylcyclohexanes and isoprenoids, only dibenzofuran was highly correlated with active ERK and IL-8 (Table S6).

PAHs and n-alkanes also showed strong correlations with the expression of aging and macroautophagy markers (Table S7, Table S8). The PAHs highly correlated with p27 were pyrene, cyclopenta[cd]pyrene, benzo[a]anthracene, benzo[b]fluoranthene, benzo[e]pyrene, indeno[1,2,3-cd]pyrene, and benzo[ghi]perylene. The macroautophagy marker, LC3B, was highly correlated with fluoranthene, pyrene, benzo[a]anthracene, benzo[b]fluoranthene, indeno[1,2,3-cd]pyrene, benzo[ghi]perylene, and coronene.

### Analysis of CMB results correlated with inflammation, aging, and macroautophagy activation

**Fig. 7:**
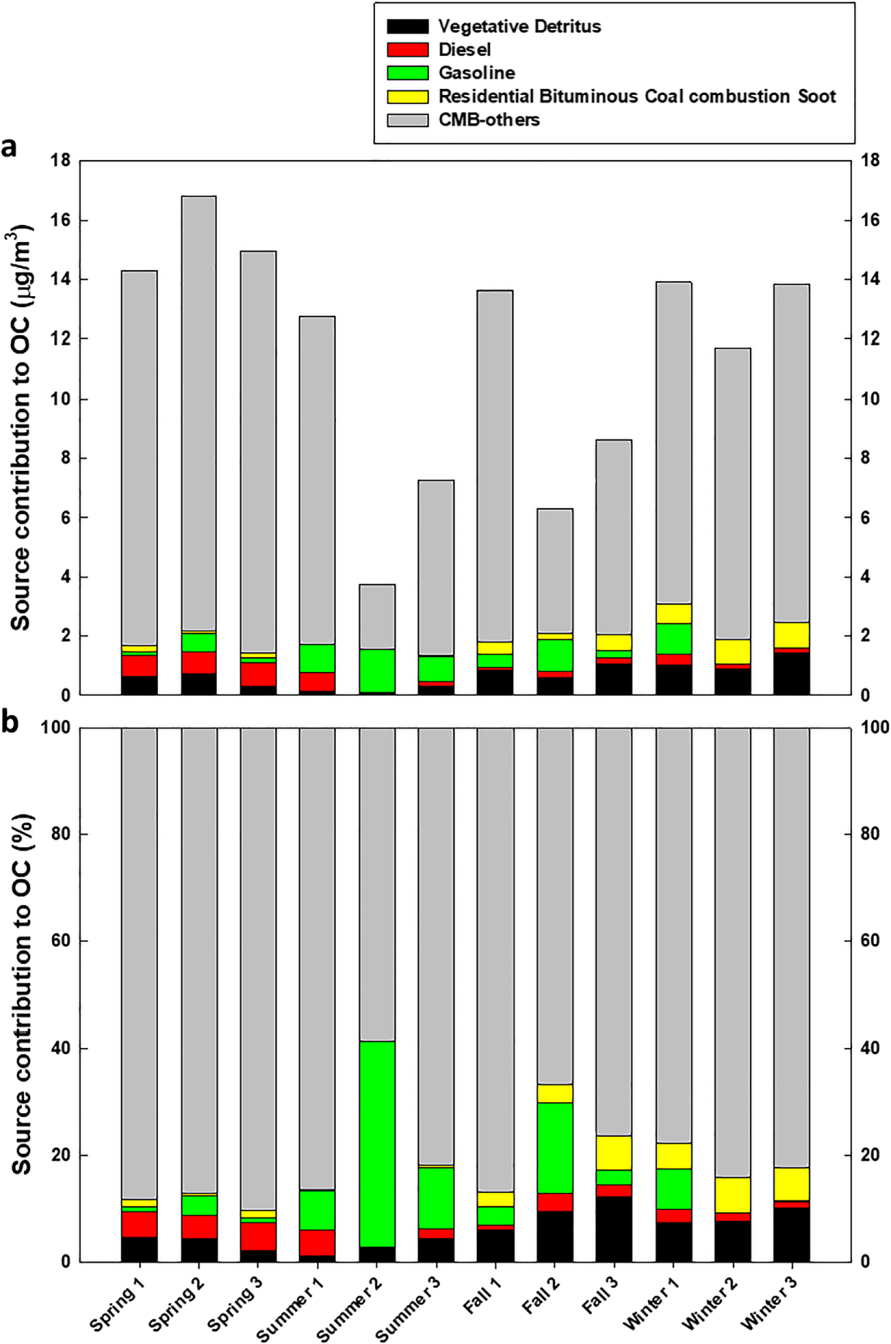
Results of the molecular marker of CMB source apportionments for the twelve samples. **a** Source contribution estimates of the four sources. **b** Percent contributions to OC of the four sources

The CMB model was employed to calculate source contributions to OC in PM_2.5_ using a molecular marker. Even though only up to 20% of organic compounds can be quantified, molecular markers have been applied for source apportionment through CMB (Schauer and Cass 2000; Zheng et al. 2002). Source contribution estimates and percentages obtained from the CMB model are displayed in Fig. 7 (Table S2). The percent contribution was calculated by dividing the source contribution estimates by the OC concentrations. Four sources were identified as major contributors: vegetative detritus, diesel engines, gasoline motor vehicles, and residential bituminous coal combustion soot.

The source with the highest percent contribution was gasoline motor vehicles (7.8%). The contribution of gasoline motor vehicles in summer (13.5%) was 11.6% higher than that in spring (1.9%). Vegetative detritus, a biogenic source from leaf abrasions (Rogge et al. 1993), had an overall average contribution of 6.0%. The contributions of vegetative detritus in fall (8.6%) and winter (8.4%) were higher than those in spring (3.6%) and summer (2.4%). Residential bituminous coal combustion soot sources had an average contribution of 2.9% to OC. The increased usage of residential heating during cold seasons may be the cause of significantly higher contributions of residential bituminous coal combustion soot in fall (4.1%) and winter (5.9%) than in spring (1.1%) and summer (0.2%). The contribution of diesel engines to the total samples was 2.8%. Although the contributions of diesel engines in spring (4.9%) and summer (3.1%) were higher than those in fall (1.9%) and winter (1.8%), the overall contributions were relatively consistent throughout the seasons. The four identified primary sources explained approximately 18% of the total PM_2.5_ source contributions; however, marked seasonal variations were observed.

**Fig. 8:**
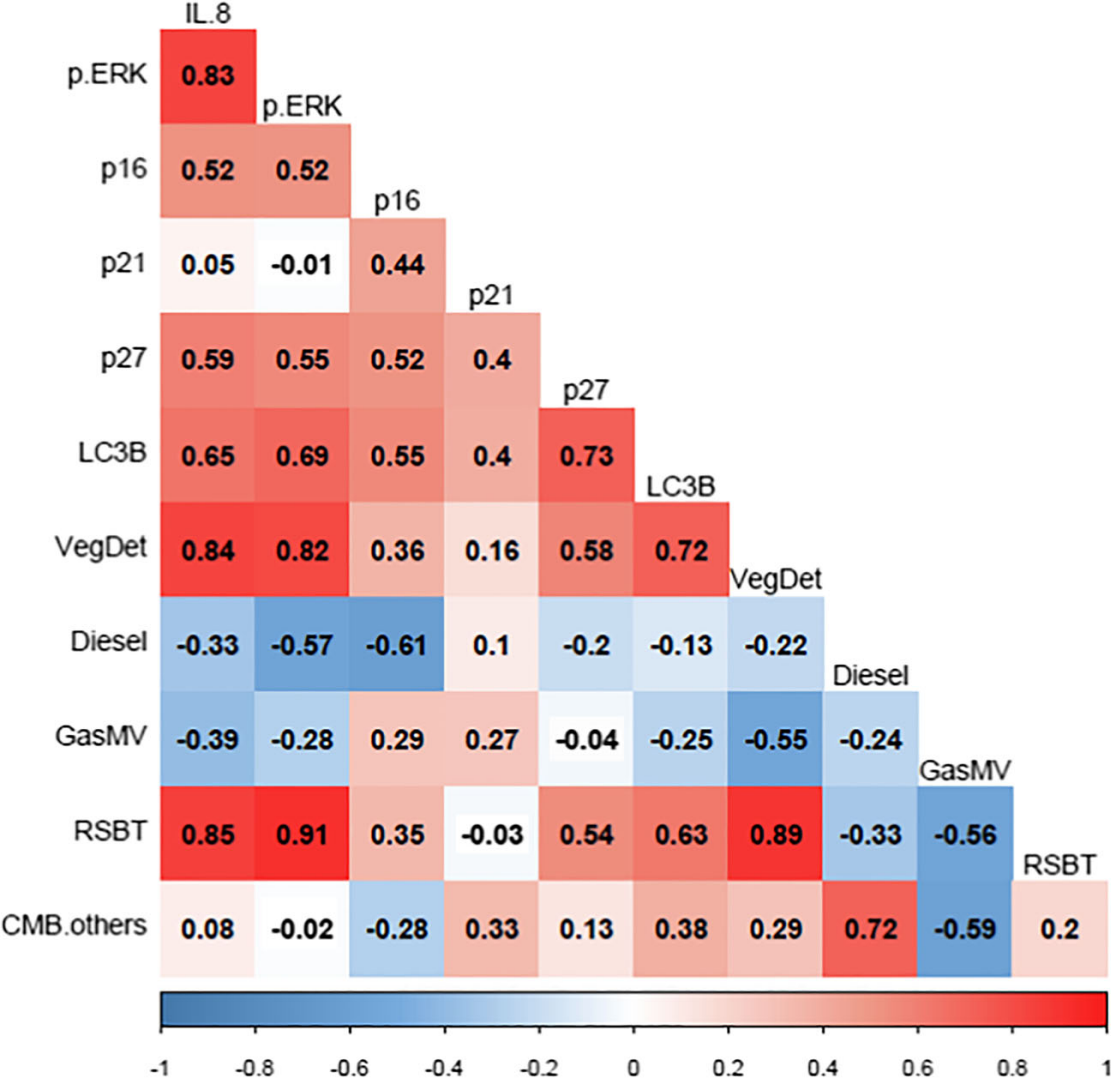
Correlation matrix between four sources and ERK activation, IL-8 production, and the expression levels of aging/macroautophagy markers (VegDet: vegetative detritus, GasMV: gasoline motor vehicles, RSBT: residential bituminous coal combustion)

Correlations among the four primary contributing sources and ERK activation, IL-8 production, and the expression levels of aging and macroautophagy markers were examined (Fig. 8); p-ERK, IL-8, p27, and LC3B showed a strong correlation with vegetative detritus and residential bituminous coal combustion. Diesel engines and gasoline motor vehicle sources did not show any significant associations. IL-8 release had strong correlations with vegetative detritus (r = 0.84) and residential bituminous coal combustion soot (r = 0.85). Similarly, ERK activation had a strong correlation with vegetative detritus (r = 0.82) and residential bituminous coal combustion soot (r = 0.91). The expression levels of p27 and LC3B had moderately strong correlations with vegetative detritus (r = 0.58 and r = 0.72, respectively) and residential bituminous coal combustion soot (r = 0.54 and r = 0.63, respectively).

## Discussion

Recently, numerous epidemiological and experimental studies have reported the effects of PM_2.5_ on lung diseases (Beelen et al. 2014; Hamra et al. 2014; Dornhof et al. 2017; Zhu et al. 2018); however, the effects of chemical components of ambient PM_2.5_ and its underlying mechanisms are still under research. Some studies have emphasized the importance of chemical components of PM_2.5_ such as PAHs and metals, but most of the exposure analyses were conducted by mixing collected PM_2.5_ samples or using commercially available SRM, which cannot accurately represent the ambient PM_2.5_ in a specific region. As toxicity of PM_2.5_ largely depends on its chemical constituents and sources, the present study focused on the impacts of organic components of ambient PM_2.5_, collected on 12 different days during HCEs in Seoul, on BEAS-2B cells and primary HAECs.

In the present study, we showed that organic extracts of PM_2.5_ collected in Seoul during HCEs induced neutrophilic inflammation, cellular aging, and macroautophagy activation in primary lung epithelial cells. In particular, several organic constituents (e.g., PAHs and n-alkanes) as well as specific sources, including biomass related sources (e.g., vegetative detritus) and residential bituminous coal combustion soot, were found to be highly correlated with increases in inflammation and cell senescence and macroautophagy activation. Senescence and macroautophagy activation in lung epithelial cells are involved in the pathogenesis of inflammatory lung diseases, such as COPD (Kuwano et al. 2016). PAHs and n-alkanes were the most relevant components to mediate ERK activation-dependent IL-8 production. IL-8 is the primary cytokine involved in the recruitment of neutrophils to the site of infection or damage (Richman-Eisenstat et al. 1993). IL-8 released from lung epithelial cells is known to recruit neutrophils to the lung, further amplifying inflammation. Mitogen-activated protein (MAP) kinases, especially ERK, play a role in PM_2.5_-induced pro-inflammatory signaling (Wang et al. 2010). The PAH compounds including benzo[a]pyrene, cyclopenta[cd]pyrene, dibenzo[a,h]anthracene, benzo[a]anthracene, benzo[b]fluoranthene, benzo[k]fluoranthene, and indeno[1,2,3-cd]pyrene significantly induced IL-8production. Moreover, the levels of aging and macroautophagy markers, such as p27 and LC3B, were found to be highly correlated with the presence of PAHs and n-alkanes. Additionally, the presence of PAHs, such as pyrene, benzo[a]anthracene, benzo[b]fluoranthene, indeno[1,2,3-cd]pyrene, and benzo[ghi]perylene, was highly correlated with the expression of p27 and LC3B.

Consistent with our results, previous studies have demonstrated that exposure to PM_2.5_ PAHs, which are major components of carbonaceous species, significantly induce pro-inflammatory cytokine production (Den Hartigh et al. 2010; Chen et al. 2019) and macroautophagy marker expression (Dornhof et al. 2017; Zhu et al. 2018). IL-8 release and ROS generation are known to be mainly related to OC, especially PAHs, i.e., the primary organic compounds obtained from heating sources. The average concentrations of PAHs were higher during cold seasons than during warm seasons in Seoul, and we found that PM_2.5_ samples from cold seasons were highly correlated with inflammation. Another study, which was conducted in Nanjing, China, reported a similar seasonal trend. Cold seasons have higher levels of PAHs, which mediate lung epithelial cell death and inflammation (Chen et al. 2019). While many studies have reported that PM_2.5_ induces the release of several inflammatory cytokines, such as IL-1β, IL-6, and TNF-α, organic extracts of PM_2.5_ collected in Seoul specifically induced IL-8 production, which might be due to the difference in chemical composition of PM_2.5_ obtained from different locations and differences in cell type.

PAHs may be emitted from both natural and anthropogenic sources. However, anthropogenically produced PAHs are predominant (Maliszewska-Kordybach 1999). Due to the relationship between temperature and vapor pressure, airborne PAHs are more likely to bind to particulate matter in winter; in contrast, larger fractions are observed in the gas phase in summer (Gualtieri et al. 2010; Holme et al. 2019). Since PAHs are produced in the process of incomplete combustion of organic materials (Kim et al. 2013), high contributions of residential bituminous coal combustion soot may have affected high concentrations of PAHs during fall and winter.

In this study, n-alkanes with a high molecular weight, such as C30 to C34, were significantly correlated with inflammation. N-alkanes are usually used as markers for sources, such as coal combustion, motor vehicle exhaust, and vegetative detritus, and are known to be related to IL-8 release and ROS generation (Perrone et al. 2013; Chen et al. 2019). In this study, vegetative detritus, which is a biogenic source, was identified using n-alkanes. However, the average carbon preference index (Tissot and Welte 1984) of the analyzed samples was 0.8, indicating the anthropogenic influence of the source.

Many epidemiological studies have discovered the association between PM_2.5_ sources and mortality (Laden et al. 2000; Ostro et al. 2011; Heo et al. 2014). In Korea, biomass burning, gasoline, and diesel emission sources have been found to be significantly associated with cardiovascular and respiratory mortality (Heo et al. 2014). Toxicological studies have determined the cytotoxicity and adverse health effects of sources, such as combustion and vehicle emission (Lippmann and Chen 2009; Diaz et al. 2012; Künzi et al. 2015; Wang et al. 2016; Velali et al. 2018; Xu et al. 2020). In this study, vegetative detritus and residential bituminous coal combustion sources were found to be highly correlated with inflammation, aging, and macroautophagy activation. No significant correlation between vehicle emission sources and inflammation and between aging and macroautophagy markers may have resulted from differences in PM_2.5_ collection methods (e.g., particles generated in a smog chamber or SRM), cell types, and receptor models (e.g., positive matrix factorization from EPA) (Künzi et al. 2015; Xu et al. 2020; Leclercq et al. 2016).

Many studies have noted that neutrophilic inflammation and macroautophagy activation are closely related to the pathogenesis of inflammatory lung diseases, such as COPD. In this study, we demonstrated that PM_2.5_ organic extracts significantly increased IL-8 production through the activation of ERK, and induced macroautophagy activation in lung epithelial cells. PAHs and n-alkanes (n-C30~n-C34), which are related to primary combustion sources, were found to be responsible for inducing inflammation and macroautophagy in lung epithelial cells. By exposing ambient PM_2.5_, we confirmed neutrophilic inflammation and macroautophagy on commercial cell line as well as epithelial cells collected from various donors. Moreover, we found out that PM_2.5_ organic extracts specifically induced IL-8 as well as macroautophagy and this is due to different chemical composition and sources that forms ambient PM_2.5_ in Seoul. Lastly, among HCEs samples, organic compounds such as PAHs and n-alkanes were found to be more important than PM_2.5_ mass concentrations itself.

## Conclusions

Organic extracts of PM_2.5_ collected in Seoul, South Korea, during HCEs induced inflammation, cellular aging, and macroautophagy activation in primary lung epithelial cells. The average mass concentrations of OC and EC had no significant correlations with PM_2.5_ effects. Both PAHs and n-alkanes were the most relevant components of PM_2.5_ for inflammation, aging, and macroautophagy activation. Our findings support the idea that the chemical constituents of PM_2.5_ are more important than the mass concentrations of PM_2.5_, and even low concentrations of PM_2.5_ may have adverse effects on public health (Feng et al. 2016; Elliott and Copes 2011; Park et al. 2018).

To the best of our knowledge, this is the first study to assess the effects of organic compounds of seasonal ambient PM_2.5_ collected in Seoul, South Korea, on inflammation, cellular aging, and macroautophagy in primary lung epithelial cells. Although the cells were not cultured at the air-liquid interface, which provides similar environment as human lungs, the exposure of PM_2.5_ organic extracts to cells collected from various donors showed similar results. Our results may be used as a reference for the implementation of PM_2.5_ reduction policy based on its chemical constituents and sources that cause adverse health effects.

There are certain limitations to this study. PM_2.5_ comprises various chemical constituents; therefore, the effects of other chemical constituents on lung epithelial cells cannot be neglected. Additionally, source apportionment using CMB with non-polar compounds can only identify primary sources; therefore, polar compounds should also be considered. Further studies that analyze other chemical constituents of PM_2.5_ using a large number of samples for detailed source apportionment are required.

## Supplementary Information

ESM 1 (PDF 383 kb)

## Data Availability

All data generated or analyzed during this study are included in this published article and its supplementary information files.
