# Supplementary material for: The impact of organic extracts of seasonal PM2.5 on primary human lung epithelial cells and their chemical characterization
Source: Environ Sci Pollut Res Int. 2021 Jun 20;28(42):59868–80. doi: 10.1007/s11356-021-14850-1 (PMC8541986; doi:10.1007/s11356-021-14850-1)
Supplement: Supplementary file 1 — (PDF 383 kb) [file 11356_2021_14850_MOESM1_ESM.pdf]

Environmental Science and Pollution Research

Supporting Information for:

The impact of organic extracts of seasonal $PM_{2.5}$ on primary human lung epithelial cells and their chemical characterization

Jieun Park[1,], Kyoung-Hee Lee[2,*], Hyewon Kim[1], Jisu Woo[2], Jongbae Heo[3,**], Chang-Hoon Lee[2], Seung-Muk Yi[1,4], Chul-Gyu Yoo[2,**]*

[1]Graduate School of Public Health, Seoul National University, Seoul, Korea

[2]Division of Pulmonary and Critical Care Medicine, Department of Internal Medicine, Seoul National University Hospital, Seoul, Korea

[3]Busan Development Institute, Busan, Korea

[4]Institute of Health and Environment, Seoul National University, Seoul, Korea

* leading author: these two authors contribute equally to this work.
**corresponding author:

Jongbae Heo, Ph.D.

E-mail address: jongbaeheo@gmail.com

Address: 955 Jungangdae-ro, Busanjin-gu, Busan, 47210, Korea

Phone: +82-51-860-8701, Fax: +82-51-860-8787

Chul-Gyu Yoo, MD

E-mail address: cgyoo@snu.ac.kr

Address: 101 Daehakno, Jongno-gu, 03080, Seoul, Korea

Phone: +82-2-2072-3760, Fax: +82-2-762-9662

CONTENTS

Table Captions

Table S1. Source Profiles for CMB 3

Table S2. Source apportionment of OC estimated by the CMB model (Unit: µg/m^3). 4

Table S3. Average concentrations for chemical species... 5

Table S4. Correlation between IL-8/active ERK and PAHs.. 6

Table S5. Correlation between IL-8/active ERK and n-alkanes.. 7

Table S6. Correlation between IL-8/active ERK and alkylcyclohexanes & Isoprenoids 8

Table S7. Correlation between p16, p21, p27, LC3B levels and PAHs 9

Table S8. Correlation between p16, p21, p27, LC3B levels and n-alkanes 10

Table S1. Source Profiles for CMB.

ID	Vegetative Detritus	Diesel Engines	Gasoline motor vehicles	Residential bituminous coal combustion
	µg/µg OC	µg/µg OC	µg/µg OC	µg/µg OC
EC	2.90E-02	2.56E+00	2.88E-01	9.24E-01
ECU	5.80E-03	2.34E-01	1.59E-02	4.62E-02
A27	2.54E-03	1.13E-04	0.00E+00	4.29E-04
A27U	5.07E-04	2.26E-05	1.00E-08	8.58E-05
A28	7.24E-04	9.52E-05	1.06E-05	1.98E-04
A28U	1.45E-04	1.90E-05	2.99E-06	3.96E-05
A29	1.84E-02	6.52E-05	1.79E-04	1.48E-04
A29U	3.68E-03	1.31E-05	5.06E-05	2.96E-05
A30	1.34E-03	0.00E+00	0.00E+00	8.55E-05
A30U	2.68E-04	1.00E-08	1.00E-08	1.71E-05
A31	2.93E-02	1.52E-04	3.56E-06	4.46E-05
A31U	5.86E-03	3.04E-05	1.01E-06	8.92E-06
A32	2.34E-03	3.97E-05	7.15E-06	2.65E-05
A32U	4.68E-04	7.94E-06	2.02E-06	5.30E-06
TNOHO	0.00E+00	6.28E-06	5.68E-05	4.37E-04
TNOHOU	1.00E-08	1.26E-06	1.14E-05	8.74E-05
B17NHO	0.00E+00	3.62E-05	1.80E-04	5.44E-04
B17NHOU	1.00E-08	7.24E-06	3.64E-05	1.09E-04
A17HOP	0.00E+00	1.65E-05	2.02E-04	2.92E-04
A17HOPU	1.00E-08	3.30E-06	4.11E-05	5.84E-05
PICENE	0.00E+00	0.00E+00	0.00E+00	7.04E-04
PICENEU	1.00E-08	1.00E-08	1.00E-08	1.41E-04
BZBFLU	0.00E+00	2.07E-05	2.29E-04	5.39E-03
BZBFLUU	1.00E-08	4.14E-06	4.65E-05	1.08E-03
BZKFLU	0.00E+00	2.90E-05	1.72E-04	1.25E-03
BZKFLUU	1.00E-08	5.80E-06	3.49E-05	2.50E-04
BZEPYR	0.00E+00	3.40E-05	3.15E-04	2.78E-03
BZEPYRU	1.00E-08	6.79E-06	6.46E-05	5.56E-04
INDPYR	0.00E+00	1.15E-07	1.83E-04	3.21E-03
INDPYRU	1.00E-08	2.93E-08	3.79E-05	6.42E-04
BZGHPL	0.00E+00	6.22E-06	6.62E-04	2.04E-03
BZGHPLU	1.00E-08	1.25E-06	1.33E-04	4.08E-04

Compound ID abbreviations

EC: Elemental Carbon
A27: Heptacosane
A28: Octacosane
A29: Nonacosane
A30: Triacontane
A31: Hentriacontane
A32: Dotriacontane
TNOHO: 17α(H)-20,29,30-Trisnorhopane
B17NHO: 17β(H)-21α(H)-30-Norhopane
A17HOP: 17α(H)-21β(H)-Hopane
PICENE: Picene
BZBFLU: Benzo(b)fluoranthene
BZKFLU: Benzo(k)fluoranthene
BZEPYR: Benzo(e)pyrene
INDPYR: Indeno(1,2,3-cd)pyrene
BZGHPL: Benzo(ghi)perylene

Table S2. Source apportionment of OC estimated by the CMB model (Unit: μg/m^3).

	VegDet		Diesel		GasMV		RSBT		R^2	X^2
	SCE	std	SCE	std	SCE	std	SCE	std		
Spring 1	0.65	0.07	0.71	0.19	0.11	0.19	0.21	0.03	0.75	6.85
Spring 2	0.73	0.08	0.73	0.18	0.63	0.17	0.10	0.03	0.74	8.49
Spring 3	0.31	0.04	0.79	0.18	0.15	0.15	0.19	0.03	0.70	9.03
Summer 1	0.15	0.03	0.62	0.18	0.94	0.17	0.01	0.02	0.67	8.11
Summer 2	0.11	0.02	0.00	0.06	1.44	0.26	-0.02	0.02	0.62	6.79
Summer 3	0.32	0.04	0.13	0.07	0.84	0.20	0.04	0.03	0.51	17.2
Fall 1	0.83	0.09	0.11	0.07	0.46	0.24	0.40	0.05	0.76	7.54
Fall 2	0.59	0.07	0.23	0.08	1.07	0.24	0.21	0.04	0.80	6.85
Fall 3	1.05	0.11	0.20	0.08	0.25	0.27	0.54	0.07	0.70	10.8
Winter 1	1.02	0.11	0.37	0.10	1.05	0.37	0.66	0.08	0.73	11.2
Winter 2	0.91	0.10	0.16	0.07	-0.51	0.34	0.80	0.09	0.73	8.12
Winter 3	1.41	0.15	0.16	0.08	0.02	0.36	0.86	0.10	0.73	9.75

VegDet: vegetative detritus; GasMV: gasoline vehicle emission; RSBT: residential bituminous coal combustion

Table S3. Average concentrations for chemical species

Chemical Species	Total (N=12)		Spring (N=3)		Summer (N=3)		Fall (N=3)		Winter (N=3)	
	mean	SEM	mean	SEM	mean	SEM	mean	SEM	mean	SEM
$PM_{2.5}$	83.23	3.85	149.10	11.19	59.03	10.10	48.36	5.97	76.42	3.41
OC	11.49	0.34	15.34	0.43	7.92	1.51	9.53	1.25	13.16	0.41
EC	1.32	0.05	2.06	0.05	0.84	0.20	1.00	0.06	1.38	0.14
ΣPAHs	18.34	1.00	9.88	0.44	7.13	0.37	22.34	2.65	34.00	2.00
Σn-alkanes	82.72	1.47	74.41	1.75	79.53	7.18	80.07	2.15	96.86	9.05
ΣHopanes	1.14	0.04	1.04	0.03	1.57	0.13	1.25	0.09	0.69	0.12
ΣAlkylcyclohexanes & Isoprenoids	62.47	4.28	0.30	0.02	0.31	0.02	0.34	0.02	0.72	0.04

Unit for $PM_{2.5}$, OC and EC is μg/m^3; Unit for organic compounds is ng/m^3

Table S4. Correlation between IL-8/active ERK and PAHs.

PAHs	IL-8		p-ERK	
	r	p value	r	p value
**PAHs sum**	**0.89**	**<0.001**	**0.92**	**<0.001**
1-Methylnaphthalene	0.06	0.845	-0.01	0.967
2-Methylnaphthalene	0.40	0.248	0.27	0.449
2,6-Dimethylnaphthalene	0.15	0.642	0.38	0.219
Fluorene	0.54	0.068	0.50	0.102
**Phenanthrene**	**0.82**	**0.001**	**0.89**	**<0.001**
**Anthracene**	**0.70**	**0.012**	**0.71**	**0.009**
**Fluoranthene**	**0.80**	**0.003**	**0.90**	**<0.001**
**Pyrene**	**0.85**	**<0.001**	**0.91**	**<0.001**
Benzo[ghi]flouranthene	0.81	0.100	0.34	0.578
**Cyclopenta[cd]pyrene**	**0.75**	**0.005**	**0.60**	**0.040**
**Benzo[a]anthracene**	**0.85**	**<0.001**	**0.83**	**<0.001**
Chrysene	0.79	0.064	0.75	0.083
**Benzo[b]fluoranthene**	**0.86**	**<0.001**	**0.90**	**<0.001**
**Benzo[k]fluoranthene**	**0.93**	**<0.001**	**0.87**	**0.002**
**Benzo[a]pyrene**	**0.90**	**<0.001**	**0.93**	**<0.001**
**Benzo[e]pyrene**	**0.89**	**<0.001**	**0.88**	**<0.001**
Perylene	0.23	0.466	0.04	0.908
**Indeno[1,2,3-cd]pyrene**	**0.88**	**<0.001**	**0.88**	**<0.001**
**Dibenzo[a,h]anthracene**	**0.86**	**<0.001**	**0.89**	**<0.001**
**Picene**	**0.86**	**<0.001**	**0.88**	**<0.001**
**Benzo[ghi]perylene**	**0.86**	**<0.001**	**0.87**	**0.001**
**Coronene**	**0.90**	**<0.001**	**0.90**	**<0.001**
Dibenz[a,e]pyrene	0.22	0.487	0.34	0.277

r>0.7 with p value<0.05 are shown in bold

Table S5. Correlation between IL-8/active ERK and n-alkanes.

n-alkanes	IL-8		p-ERK	
	r	p value	r	p value
n-alkanes sum	0.36	0.248	0.59	0.044
n-C21	-0.12	0.880	0.95	0.054
n-C22	0.12	0.848	0.79	0.115
n-C23	0.10	0.871	0.81	0.100
n-C24	0.15	0.806	0.87	0.053
n-C25	0.41	0.420	**0.92**	**0.010**
n-C26	-0.49	0.181	-0.22	0.575
**n-C27**	**0.87**	**<0.001**	**0.92**	**<0.001**
n-C28	0.27	0.512	0.49	0.219
n-C29	0.36	0.252	0.22	0.498
**n-C30**	**0.80**	**0.002**	**0.76**	**0.004**
**n-C31**	**0.93**	**<0.001**	**0.83**	**<0.001**
**n-C32**	**0.91**	**<0.001**	**0.84**	**<0.001**
**n-C33**	**0.93**	**<0.001**	**0.89**	**<0.001**
**n-C34**	**0.83**	**<0.001**	**0.80**	**0.002**
n-C35	-0.38	0.225	-0.19	0.549
n-C36	-0.71	0.009	-0.80	0.002
n-C37	-0.10	0.760	-0.03	0.915

r>0.7 with p value<0.05 are shown in bold

Table S6. Correlation between IL-8/active ERK and alkylcyclohexanes & Isoprenoids.

Alkylcyclohexanes & Isoprenoids	IL-8		p-ERK	
	r	p value	r	p value
Alkylcyclohexanes and Isoprenoids sum	-0.31	0.328	-0.31	0.324
**Dibenzofuran**	**0.61**	**0.036**	**0.76**	**0.004**
9-Methyl-fluorene	0.34	0.278	0.49	0.108
2-Methylnonadecane	0.17	0.595	0.26	0.423
3-Methylnonadecane	0.20	0.529	0.30	0.340
Nonadecylcyclohexane	-0.46	0.129	-0.76	0.005

r>0.6 with p value<0.05 are shown in bold

Table S7. Correlation between p16, p21, p27, LC3B levels and PAHs.

PAHs	p16		p21		p27		LC3B	
	r	p value	r	p value	r	p value	r	p value
**PAHs sum**	0.50	0.100	0.17	0.606	**0.75**	**0.005**	**0.71**	**0.009**
1-Methylnaphthalene	0.19	0.557	-0.43	0.162	-0.21	0.512	-0.12	0.700
2-Methylnaphthalene	0.37	0.297	0.07	0.842	0.04	0.908	0.32	0.363
2,6-Dimethylnaphthalene	0.06	0.854	0.00	0.997	0.51	0.088	0.20	0.539
Fluorene	**0.69**	**0.012**	0.05	0.871	0.46	0.129	0.46	0.138
Phenanthrene	0.50	0.095	0.22	0.486	**0.69**	**0.014**	**0.69**	**0.013**
Anthracene	**0.58**	**0.047**	0.36	0.250	**0.65**	**0.022**	0.54	0.069
Fluoranthene	0.35	0.299	0.14	0.677	**0.68**	**0.020**	**0.72**	**0.012**
Pyrene	0.44	0.150	0.20	0.532	**0.74**	**0.006**	**0.72**	**0.008**
Benzo[ghi]flouranthene	-0.20	0.749	0.22	0.728	0.74	0.149	0.52	0.374
Cyclopenta[cd]pyrene	0.44	0.150	0.47	0.127	**0.81**	**0.001**	**0.63**	**0.029**
Benzo[a]anthracene	0.57	0.052	0.37	0.243	**0.77**	**0.003**	**0.71**	**0.010**
Chrysene	-0.21	0.697	0.47	0.350	0.72	0.107	0.74	0.093
Benzo[b]fluoranthene	0.44	0.150	0.17	0.591	**0.70**	**0.011**	**0.71**	**0.009**
Benzo[k]fluoranthene	0.40	0.282	0.01	0.979	0.61	0.081	0.56	0.120
Benzo[a]pyrene	0.51	0.092	0.14	0.666	**0.67**	**0.016**	**0.66**	**0.019**
Benzo[e]pyrene	0.49	0.109	0.16	0.620	**0.71**	**0.010**	**0.65**	**0.022**
Perylene	-0.21	0.520	0.28	0.373	0.34	0.279	0.03	0.926
Indeno[1,2,3-cd]pyrene	**0.63**	**0.049**	0.44	0.200	**0.78**	**0.008**	**0.85**	**0.002**
Dibenzo[a,h]anthracene	0.42	0.176	0.13	0.693	**0.68**	**0.014**	**0.66**	**0.021**
Picene	0.43	0.161	0.14	0.673	**0.69**	**0.014**	**0.65**	**0.022**
Benzo[ghi]perylene	0.56	0.095	0.29	0.416	**0.71**	**0.022**	**0.73**	**0.017**
Coronene	0.50	0.096	0.24	0.445	**0.67**	**0.018**	**0.73**	**0.007**
Dibenz[a,e]pyrene	0.00	0.999	-0.23	0.464	0.40	0.203	0.34	0.287

r>0.5 with p value<0.05 are shown in bold

Table S8. Correlation between p16, p21, p27, LC3B levels and n-alkanes.

n-alkanes	p16		p21		p27		LC3B	
	r	p value	r	p value	r	p value	r	p value
n-alkanes sum	-0.06	0.851	-0.05	0.883	0.47	0.120	**0.65**	**0.022**
n-C21	-0.80	0.204	-0.62	0.379	-0.01	0.990	0.56	0.443
n-C22	-0.69	0.198	-0.22	0.718	0.33	0.587	0.66	0.227
n-C23	-0.80	0.104	-0.39	0.521	0.19	0.759	0.55	0.340
n-C24	-0.78	0.118	-0.41	0.497	0.21	0.738	0.57	0.315
n-C25	-0.65	0.160	0.02	0.975	0.37	0.470	0.67	0.143
n-C26	-0.16	0.683	-0.21	0.583	0.15	0.699	0.11	0.787
n-C27	0.35	0.271	0.05	0.888	**0.59**	**0.044**	**0.75**	**0.005**
n-C28	-0.44	0.277	-0.09	0.830	0.10	0.806	0.48	0.233
n-C29	0.09	0.772	0.26	0.417	0.39	0.213	0.44	0.155
n-C30	0.39	0.212	0.27	0.401	0.57	0.052	**0.71**	**0.009**
n-C31	**0.72**	**0.009**	0.13	0.689	**0.65**	**0.021**	**0.68**	**0.015**
n-C32	0.51	0.088	0.22	0.484	**0.67**	**0.018**	**0.69**	**0.013**
n-C33	**0.63**	**0.027**	0.12	0.722	**0.67**	**0.017**	**0.67**	**0.017**
n-C34	0.47	0.127	0.11	0.727	**0.60**	**0.039**	**0.60**	**0.038**
n-C35	-0.23	0.482	-0.09	0.781	-0.14	0.661	-0.07	0.830
n-C36	-0.59	0.043	-0.12	0.706	-0.54	0.071	-0.56	0.057
n-C37	-0.08	0.802	-0.31	0.321	0.00	0.993	0.09	0.787

r>0.5 with p value<0.05 are shown in bold
